# Microfluidic Ecology Unravels the Genetic and Ecological Drivers of T4r Bacteriophage Resistance in E. coli: Insights into Biofilm-Mediated Evolution

**DOI:** 10.21203/rs.3.rs-4356333/v1

**Published:** 2024-05-24

**Authors:** Krisztina Nagy, Sarshad Koderi Valappil, Trung V. Phan, Shengkai Li, László Dér, Ryan Morris, Julia Bos, Sophia Winslow, Peter Galajda, Gábor Ràkhely, Robert H. Austin

**Affiliations:** 1 Institute of Biophysics, HUN-REN Biological Research Centre, Szeged, Hungary; 2 Department of Biotechnology, University of Szeged, Szeged, Hungary; 3 Department of Chemical and Biomolecular Engineering, Johns Hopkins University, Baltimore, MD, USA; 4 Department of Physics, Princeton University, Princeton, NJ, USA; 5 School of Physics & Astronomy, University of Edinburgh, Edinburgh, Scotland; 6 Institut Pasteur, Université Paris Cité, CNRS UMR 3525, Unité Plasticité du Génome Bactérien, Paris, France; 7 University of Northwestern St. Paul, Roseville, MN, USA

## Abstract

We use a microfluidic ecology which generates non-uniform phage concentration gradients and micro-ecological niches to reveal the importance of time, spatial population structure and collective population dynamics in the de *novo* evolution of T4r bacteriophage resistant motile *E. coli*. An insensitive bacterial population against T4r phage occurs within 20 hours in small interconnected population niches created by a gradient of phage virions, driven by evolution in transient biofilm patches. Sequencing of the resistant bacteria reveals mutations at the receptor site of bacteriophage T4r as expected but also in genes associated with biofilm formation and surface adhesion, supporting the hypothesis that evolution within transient biofilms drives *de novo* phage resistance.

## 1. Introduction

Bacteriophages (phages) are viruses that infect bacteria. Phages coexist with microbes, playing a fundamental role in microbial diversity, population dynamics and evolution. Understanding the interaction between phages and bacteria gives us fundamental information on ecological and evolutionary processes [1]. Phages can be divided into two classes: temperate and virulent. The life history for temperate phages typically consists of two parts: a lysogenic phase after inserting their DNA into the genome of a host bacterium after infection, and a propagating lytic phase where they extract their genome from the host genome, reproduce within the bacterium and exit by lysis of the bacterium. Virulent phages only have a lytic cycle; they do not insert their DNA into the host but rather use the cell’s machinery to make copies and lyse the cell.

Bacteriophage T4 can pause cell lysis in super-infected bacteria to yield very high titer yields in the lysis of remaining bacteria. The T4 phage strain used in this study is highly virulent and rapidly lyses the cell after infection (it is an obligate lytic, [2]). Phage T4r (T4 rapid) lacks the lysis inhibition (LIN) genes of wild-type T4 and rapidly lyses bacteria, even multiply infected “super-infected” bacteria, with resultant relatively low titer yields compared to wild-type T4 [3]. Because of the rapid lysis of T4r infected bacteria any survival of a low number *N*_*O*_ of bacteria can only be due to either previously acquired resistance (Darwinian random mutations) or a kind of rapid response by the stress-challenged bacteria, which can be called Lamarckian in a certain sense of the word.

In the 1940s the pioneering studies of Luria and Delbrück [4] explored phage resistance to the obligate lytic phage T1 in *E. coli* using a protocol of well-mixed culture flasks and plaque formation assays on agar. These studies were interpreted to show that bacterial mutations giving resistance occurred in the absence of selective pressure rather than being a response to it, contradicting the Lamarckian acquired evolution hypothesis which at the time suggested that specific mutations are acquired specifically in response to certain environmental stresses. Luria and Delbrück proved that pre-existing mutations conferring resistance were present in a sufficiently large bacterial population. However, they did not disprove the possibility that acquired resistance in response to stress [5] might also occur and contribute to the emergence of resistance [6]. Furthermore, the homogeneous low-stress environment of a well-mixed culture flask does not resemble the complex ecologies in which bacteria typically grow, and they do not allow for bacterial motility across ecological regions with differing stress levels and the spread of resistance from *de novo* mutational response to phage infection [7].

Since then, we have learned that mutations in response to stress do occur [8] and that there are different ways for organisms from bacteria to man to acquire heritable phenotypic changes upon transient exposure to stress [9, 10]. We also know that both genome-wide mutation rate and the mutation rates of specific genes can be affected by the environment [11–13]. Furthermore, the complexity of the environment (e.g., compartmentalization) might facilitate the fixation of mutations in bacterial populations [14, 15]. In the past decade more and more papers highlighted the importance of spatial variations in antibiotic concentration gradients in the evolution of bacterial antibiotic resistance [16–19]. We have shown in a previous publication that stress gradients imposed over a metapopulation of weakly coupled communities can greatly increase the rate at which resistance evolved to the mutagenic antibiotic ciprofloxacin [18].

In this study, we examine how stress gradients have an impact on the emergence of phage resistance in small interconnected *E. coli* populations with time. Bacteria can develop resistance *de novo* against phage infections in different ways: by gene mutations and by an ”adaptive immune response” using the CRISPR-Cas system to detect and destroy DNA from similar viruses during subsequent infection [20,21]. Laboratory strains of *E. coli* possess a functional CRISPR-Cas system, however, this system seems to be ”silent” under normal laboratory conditions [22, 23]. Although *E. coli* is an important model organism for the characterization of the CRISPR-Cas systems, and detailed descriptions of its structure and molecular mechanism in this species can be found in the literature [24–26], the physiological conditions needed for its activation is still unclear and it needs to be further investigated.

For a realistic bacterial-phage scenario it is important to try and replicate the natural habitats of microorganisms, which are complex - the environments have physical heterogeneity and heterogeneous distribution of resources. However, most studies have been carried out in well-mixed populations or chemostats [27] or on agar plates with homogeneous phage concentrations. These studies do not capture some important complexities of natural communities (e.g. spatial [28] and temporal [29] heterogeneity). Microfluidics provide excellent tools to mimic such aspects of natural habitats and study questions related to microbial ecology [30]. For example, a microfluidic mother machine study revealed the importance of the presence of spatial refugees in an *E. coli* population against bacteriophage T4 in structured environments compared to well-mixed cultures [31]. They suggest that structured environments promote the selection of phenotypic variants with low phage receptor expression.

In this paper, we aim to shed light on the fundamental processes behind the evolution of bacteriophage resistance. We use a microfabricated environment in which we exposed motile *E. coli* bacteria to spatial gradients of T4 bacteriophage T4r (The “r” in T4r denotes a mutation that leads to rapid lysis of the bacterial host) which reproduce by the lytic cycle when infecting *E. coli*. The growth and distribution of the bacterial population were followed in time by fluorescence time-lapse microscopy. Resistant cells were collected from the microfluidic device for further analysis, e.g., genomic sequencing was performed to identify key mutations leading to the observed resistance.

## 2. Methods

### Culturing bacteria and phage

Bacteriophage T4r (Carolina Biological Supply Company) and *E. coli* AD62 strain were used in the experiments. AD62 is derived from the K-12 strain AB1157 [32] that has the λ-deficiency, which makes it a suitable host for the T4r phage we used in our experiments. The *E. coli* strain was transformed with the pWR21 plasmid [33] for constitutive green fluorescent protein (eGFP) expression. The bacteriophage concentration was 2×10^9^ virus particles/ml in the experiments. The proper phage concentration was measured by standard PFU assay.

Before each experiment bacteria were grown overnight in plastic tubes using 2 ml lysogeny broth (LB) medium supplemented with 100 *μ*g/ml ampicillin at 37 °C in an incubator shaker (200 rpm). Overnight cultures were diluted back in the morning, and cells at a concentration of OD600=0.6 (optical density measured at 600 nm) were used for the inoculation of the microfluidic device. The number of N bacteria/ml at OD600=0.6 is approximately 2×10^8^/ml determined by CFU assay. The volume of the center well of the device is approximately 4×10^−2^ *μ*l. Thus, we predict and can confirm that we inoculated the center well with approximately *N*_*i*_ = 10^4^ bacteria at *t* = 0.

After the experiments resistant cells were isolated by opening the device and using the silicon part for replica plating on agar plates that were previously coated by 10^9^ particles of T4r [4, 34]. The selection plates with the imprints of the device were incubated overnight (16 *h*) at 37 °C. Colonies were picked from the regions of the imprint where resistant growth was observed and further analyzed. The key parameter is the spontaneous rate of single-nucleotide polymorphisms in unstressed *E. coli* Θ_*D*_ ≈ 2.0×10^−9^/generation [35].

Optical density measurements (at 600 nm) were carried out to characterize the growth of the wild type *E. coli* strain and the mutants isolated from the experiments. The optical density was measured in 110 *μ*l volumes in 96-well plates by using a BioTek Synergy H1 microplate reader. Overnight cultures (2 ml LB, plastic tubes, 200 rpm, 37 ^°C^) were backdiluted in the morning 500 times. When the optical density of the cultures reached 0.6, 100 *μ*l of the bacterial cultures (with or without dilution) together with 10 *μ*l of bacteriophage solution (with the appropriate phage concentration) were measured into the wells. The duration of the plate reader experiments were 24 hours, the well plate was shaken continuously (double orbital, 425 cpm frequency), and the temperature was set to 37 °C. Optical density was measured every 5 minutes.

The surface-adhered biofilm-forming ability of the ancestral and mutant strains was tested using the microtiter plate biofilm assay (based on crystal violet staining) [36]. The assay was performed in a 96-well plate. 100 *μ*l of cell cultures (OD=0.6, in LB) were loaded in each well and incubated for 48 hours at 37 °C without phage. Three replicates were made for each sample.

### Microfluidic device setup

**Figure 1.**
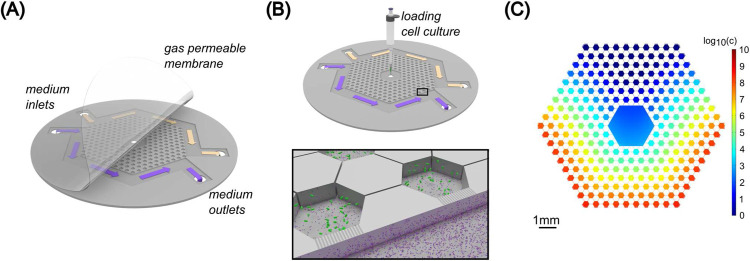
Microfluidic setup. A) 3D drawing of the microfluidic device (not-to-scale). The etched silicon chip is sealed with a 25 *μ*m thick gas permeable LUMOX film, pressurized from the front. Arrows indicate the direction of medium flow. Yellow color is used for pure LB (top channel) and the purple color corresponds to LB supplemented with T4r phages (bottom channel). B) Mounting of the chip in a LUMOX dish with applied external sealing back air pressure. Bacteria are inoculated into the middle inlet hole with a pipette. The inset shows the phage (purple particles) gradient forming from the bottom channel. Shallow (100 nm deep) nanoslits connect the side channels and the outer hexagon chambers. C) Simulation of the phage gradient present in the device at the initial stage of the experiment. Phage concentration c is indicated by the colorbar in logarithmic scale. The unit of c is virion/ml.

We used the basic hexagon array of gradient microhabitats used in [18]. The microfluidic device was etched in two layers into a silicon wafer. The schematic representation of the device is presented in Fig. 1. It is a network of hexagonal wells that are connected through narrow channels etched to 10 *μ*m depth. The nutrient supply of this network is provided from two side channels that are connected to the outer wells through 100 nm deep nanoslits. The total etched area of the hexagon array is approximately 40 mm^2^.

The top of the etched device is reversibly sealed by a 25 *μ*m thick gas-permeable Lumox membrane (SARSTEDT AG & Co. Nümbrecht, Germany). Sealing of the device top was done by pressurizing the outside of the structure with atmospheric composition air at 2×10^4^ Pa. This sealed the film against the silicon wafer but did not close the 100 nm deep etched nanoslits of the device. However, as we discuss in the text, the finite pressure of the sealing film sometimes allowed bacteria to form highly condensed colonies by pushing the film up.

Medium mixed with phage flow was provided by syringe pump at 5 *μ*l/h throughout the experiments. Before each experiment, the chip was run with LB medium (supplemented with 100 *μ*g/ml ampicillin) and bacteriophage T4r in one of the side channels (always the bottom one in the images) for 20 hours to ensure an initial virus gradient and nutrient concentration within the device. The highest phage concentration applied in the side channel was 2×10^9^ virus particles/ml. The concentration of bacteriophage within the microfluidic device was calculated for a full 3D model with COMSOL Multiphysics 4.3a software (COMSOL AB, Stockholm, Sweden) over the timescale of the experiment. The diffusion constant of the phage was estimated to be 8×10^−8^cm^2^/s [37,38]. Bacteria were inoculated into the middle of the device with a pipette at 10^4^ cell number into the 0.04 *μ*l volume of the inlet hole. Experiments (three biological replicates) were carried out at 30 °C.

### Image acquisition

Fluorescence time-lapse microscopy was performed by using a Nikon TE2000-E inverted microscope and a Canon camera (EOS5d). To get a high-resolution image series, in one experiment we used a Nikon 90i upright microscope supplemented with an ANdor Neo 5.5 sCMOS camera and a 4X Plan APO λ objective. Figures in the paper were prepared using the high-resolution image series. In both setups, a GFP filter set was used to monitor the growth and spatial distribution of bacterial populations within the device. *μ*Manager and NIS Elements software were used to take images every 15 minutes.

### Whole genome sequencing

Resistant cells were collected after the experiments on selection plates and clones were further analyzed by whole genome sequencing (The Sequencing Centre, Fort Collins, CO 80524, USA). Total DNA was extracted by using the Zymo Research Quick-DNA Fungal/Bacterial Microprep Kit (Catalog No. D6007) according to the manufacturer’s instructions. The Illumina (illumina.com) Nextera XT DNA Library Prep Kit was used to prepare extracted bacteria DNA for sequencing. Bacterial whole genome sequencing was performed on an Illumina MiniSeq short-read sequencer using a standard Illumina workflow and configured for 2 × 150 bp paired-end reads and MiniSeq flow cell.

The sequenced samples included one clone of the ancestral strain and three clones isolated from the experiments. We identified mutations related to T4 phage resistance and examined whether the CRISPR-Cas defense mechanism of *E. coli* -that is mostly repressed in lab cultures [39] - was activated under such circumstances. The raw sequences have been deposited under the study number PRJEB73316 at the European Nucleotide Archive (ENA). Paired-end sequencing with 150 bp read length was used to generate reads. Quality assessment was carried out with FastQ [40]. We trimmed raw reads to remove adapter sequences and PhiX174 contamination using BBduk. Sequence reads were assembled using Unicycler (version v0.4.7) [41]. The assembled sequences were annotated with Prokka (version 1.14.6) [42]. BBMap utilities were applied to obtain assembly metrics’ statistics [43, 44]. The annotated genomes were compared to identify the missing genes across the strain and mutants using Roary [45]. We identified SNPs in the mutants by mapping the reads to the wild-type strain using Breseq (version: 0.35.4) [46].

## 3. Results

### Colonization of the microstructured habitat by motile bacteria in the presence of phage gradient

A microfabricated landscape was used to study the evolution of bacteria against lytic phages. The microfluidic device consisted of a network of hexagon microchambers that were connected through narrow corridors (see Methods). Nutrient supply was ensured by a constant flow of fresh LB medium in the side channels that were connected (by nanoslits) to the outer patches (Fig. 1A–B). Motile *E. coli* cells were inoculated into the inlet hole positioned at the center of the device and can move between the microchambers and find the most favorable conditions. In the absence of any phage gradients, bacteria form chemotactic waves [47] and move over to the nanoslits which act as nutrient sources in this system. This rapid chemotaxis (which takes a couple of hours) towards the nanoslits occurs because of the extremely small volume (approximately 0.4 *μ*l) of medium within the hexagon array, and the rapid local consumption of nutrients by bacteria.

In our experiments, we set up an initial T4r phage gradient across the hexagon array, thus the inoculated bacteria were immediately in contact with lytic T4r phage. The initial distribution of phages in the microfluidic device, in the case of flowing LB medium supplemented with phages (2×10^9^ virus particles/ml concentration) in the bottom side channel, was estimated by a 3D model and is presented in Fig. 1C. Based on our simulations, this is not a steady state, the gradient slowly changes throughout the experiment. Also, the lysis of bacteria infected by phage disturbs the phage gradient in the later phase of the experiments. The local increment of phage concentration upon lysis events is not included in the 3D model, but it represents well the initial microenvironment.

**Figure 2.**
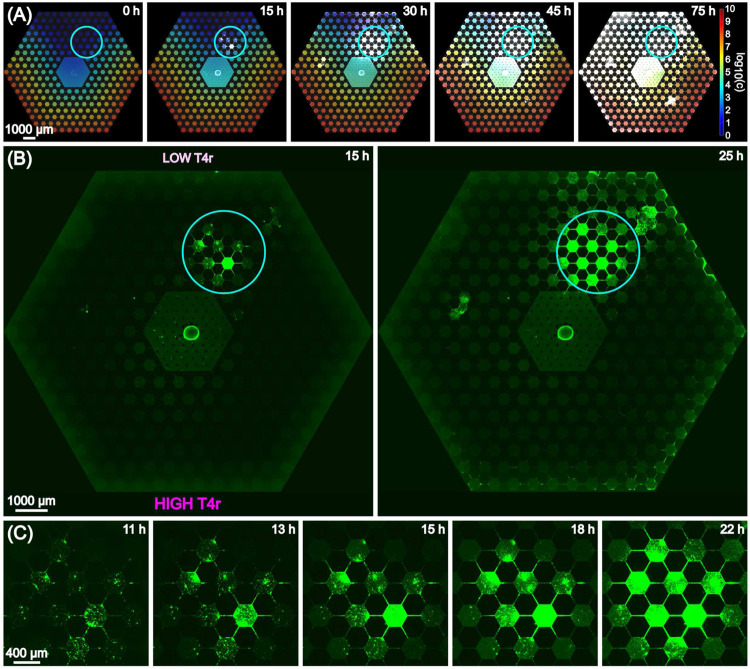
Progression of bacterial growth in T4r phage gradient. The emergence of an insensitive population at the low-phage concentration side of the device is outlined by a solid circle. (A) Snapshots of fluorescence microscopy images overlaid on the calculated T4r concentration gradient over a period of 75 hours. Phage concentration (c) is represented in logarithmic scale. The unit of c is virion/ml. (B) Stitched image of the full microfluidic array from 15 hours to 25 hours after inoculation. The solid blue circle outlines the hot spot of the insensitive bacterial population against T4r phage. (C) Emergence of insensitive sub-population from small clusters of bacteria over a time scale from 11 hours to 22 hours

Fig. 2 shows the basic progression of motile *E. coli* in the device. In the case of T4r phage gradient the previously mentioned fast chemotactic waves were lacking. Instead, localized emergence of insensitive sub-populations at low-intermediate phage concentrations within 24 hours was the typical response we observed. Fig. 2A shows fluorescence snapshots overlaid on top of the calculated concentration profile over the 75-hourlong experiment. Supplementary movie 1 contains the entire time-lapse image series of the experiment. The ”hot-spot”, which refers to the location where the insensitive population starts intense growth and colonization, is outlined by the solid circle in Fig. 2A,B, whereas Fig. 2C shows zoom-in images of this region. We can see aggregation of cells and the formation of small clusters from which bacteria spread to other parts of the device as well Fig. 2B,C. Note, that in Fig. 2B it can be seen that after 25 hours single cells also appear at the nanoslits even at the high phage concentration side of the hexagon array.

**Figure 3.**
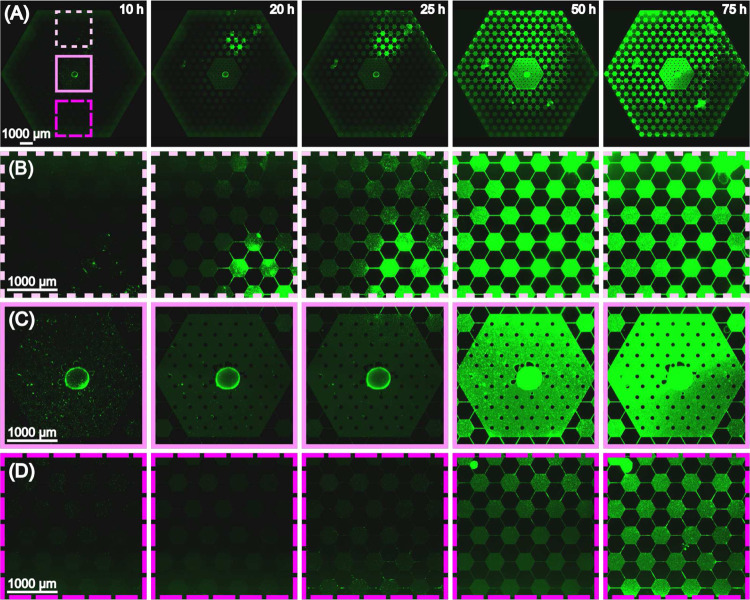
(A) Stitched images of the full array from 10 hours to 75 hours after inoculation. The dashed and solid squares outline regions of the habitat with different phage concentrations. (B) Zoom-in images of the top region of low T4r density.(C) Zoom-in images at the inoculation region in the center of the device. (D) Zoom-in images of the bottom region of high T4r density.

The observed progression pattern is the result of different mechanisms: (1) chemotaxis towards the nutrient sources, (2) response to the stress exposed by phage. The latter one might imply e.g. producing extracellular materials, biofilm formation or the appearance of stress-induced mutations in the complex environment. The details of the progression is presented in the fluorescence images of Fig. 3 by zooming into the different regions (with different phage concentrations) of the microarray habitat over the time scale of an experiment (same as presented in Fig. 2). Fig. 3 shows that after 10 hours there are bacteria in the outer hexagon chambers (right next to the nanoslits). Bacteria spread fast on the phage-free/low-phage concentration side of the habitat. However, cells (mostly single cells) can be detected on the high phage concentration side as well within 10 hours. Fig. 3A and Supplementary Movie 1 shows that 48 hours is enough for the population to colonize the the whole habitat. However, black areas can be clearly identified in the landscape (Fig. 3A) that probably represent regions of lyzed cells as a consequence of local phage infections. Occasionally, bacteria form such dense biofilm-like structures that slightly lift the sealing membrane. This was not observed before when using the same setup in evolution studies against antibiotics [18].

### Characterization of bacteria extracted from the microarray habitat

Three biological replicates were performed and the dynamics of the bacterial populations in the bacteriophage stress landscape were followed over 3–4 days. In all cases, the insensitive sub-populations appeared within a day and the basic progression pattern was very similar to what is described and presented in Fig. 3. After each experiment, the device was taken apart to collect cells. For this purpose, the Lumox cover of the ecology was removed from the silicon and resistant clones were isolated using selection plates (see Methods). Three colonies were randomly selected for further analysis. The growth properties and whole-genome sequencing data were compared to the ancestor *E. coli* strain (see Methods).

The results of whole-genome sequencing confirmed the presence of mutations related to phage resistance in the cells that were exposed to the T4r phage gradient during the experiments. The relevant mutations are summarized in Table 1.

The most obvious mutations related to T4r resistance occurred at the receptor site of bacteriophage T4 (*ompC*) [48–50]. Two out of the examined three isolates (mutant1 and mutant3 in Table 1) had changes in this particular gene, which could serve as a primary defence mechanism by inhibiting phage adsorption. The other detected mutations could also contribute to the reduced entry of the phage into the cell, and most of them can be associated with the adhesion properties and biofilm-forming abilities of *E. coli*. E.g., the rfa locus is important in the barrier function of the outer membrane. *rfaP* is involved in the pathway of the LPS core biosynthesis [51]. Besides *rfaP*, mutations were detected in *csgB*, *csgD*, *rcsC*, which are important in the normal biofilm formation of *E. coli* [52, 53]. In two samples (mutant1 and mutant2) both *csgB* and *csgD* were altered. These genes have a crucial role in the expression of curli fimbrae and possess a positive role in biofilm formation [54]. *rcsC* is associated to mucoid phenotype and to normal biofilm formation on solid surfaces [55]. It is also involved in colanic acid synthesis. Indeed, mutant2 having an SNP in *rcsC*, has a mucoid phenotype when forming colonies on a hard agar surface. The sequencing results together with the time-lapse images (Fig. 2 and Fig. 3) suggest that biofilm formation has a crucial role in bacterial phage resistance.

In the past decade, the role of clustered regulatory interspaced short palindromic repeats (CRISPR) and the associated *cas* genes in resistance against phages got acknowledged [20]. The activity of the CRISPR system of *E. coli* had not been reported previously under normal laboratory conditions. However, we were curious if the structured landscape we designed could somehow induce it. For this purpose, we compared the CRISPR regions of the isolates to the ancestor strain. No changes could be detected in the spacer sequences.

**Figure 4.**
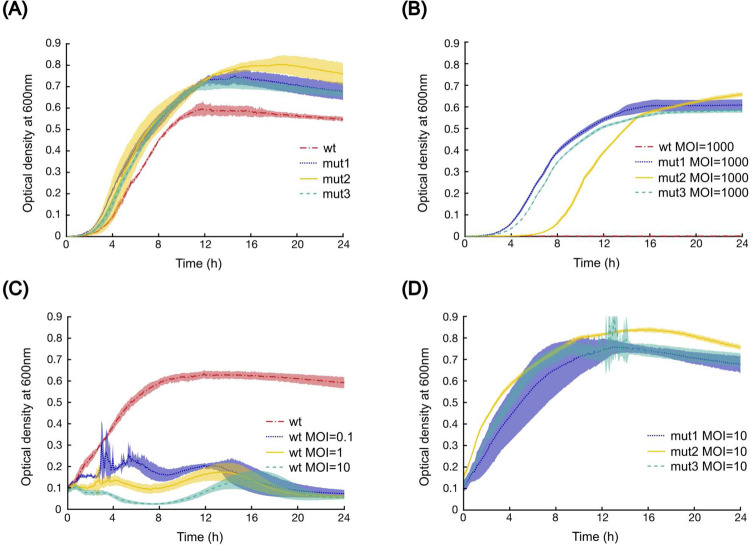
Growth properties of the ancestor and three mutant *E. coli* strains. (A) Growth of the ancestor (red dashed line) and three mutant (mut1: dotted blue line; mut2: continuous yellow line; mut3: dashed green line) strains in phage-free LB media. (B) Growth of the ancestor (red dashed line) and 3 mutant (mut1: dotted blue line; mut2: continuous yellow line; mut3: dashed green line) strains in the presence of high phage concentration (MOI=1000, the initial cell number is 100000). (C) Growth of the ancestor strain in LB media at different MOI. T4r was added to mid-log phase bacteria culture, the initial bacteria cell number is 10000000. (D) Growth of the three mutant strains when applying phage (MOI=10) at mid-log phase culture. The initial cell number is 10000000 (mut1: dotted blue line; mut2: continuous yellow line; mut3: dashed green line).

The growth properties of the mutants in the presence and absence of T4r were compared to the ancestor strain by measuring the optical density of cell cultures in 96-well plates (see Methods). Fig. 4A shows that there was no apparent fitness cost to achieving T4r resistance. The growth curves of the mutants are similar regardless of the presence of the phage (Fig. 4A,B).

The high-level resistance against T4r is shown in 4B, where the bacteria-phage cultures were started by applying MOI=1000. Under such circumstances, the growth of the ancestor strain was completely excluded. Addition of T4r to a growing culture of the ancestor bacterial strain at mid-log phase at lower MOI values (from 0 to 10) results in no change to the further growth of the resistant strain but the lysis of wild-type bacteria, as shown in Fig. 4C,D.

**Figure 5.**
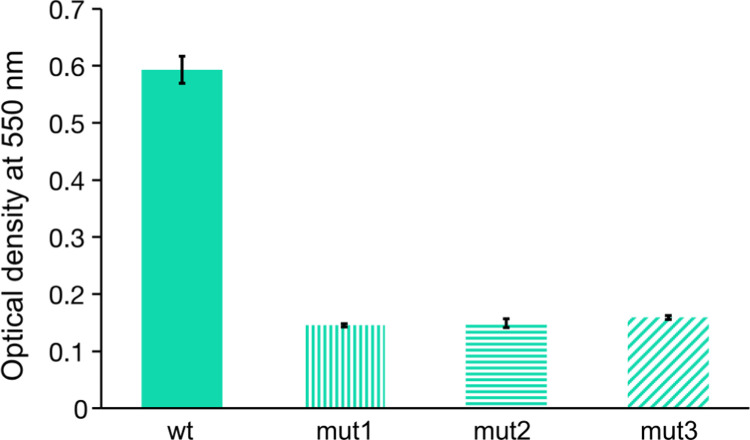
Biofilm forming ability of the ancestral and the mutant strains evolved in the microfluidic device in the presence of bacteriophage T4r gradient. The applied crystal violet staining shows the amount of surface-adhered biofilm after 48 hours of incubation at 37°C. Three replicates were performed for each sample.

The presence of mutations in *csgB*, *csgD* and *rcsC* genes, suggest that the mutants show differences in biofilm-forming and surface adhesion properties compared to the ancestor strain. Therefore, we probed the mutant strains for their ability to form surface-adhered biofilms using the crystal violet staining protocol [36]. Interestingly, as shown in Fig. 5, the mutant strains had substantially reduced biofilm-forming ability, indicating that the reduction in phage sensitivity also resulted in reduced ability to form surface-adhered biofilms.

**Figure 6.**
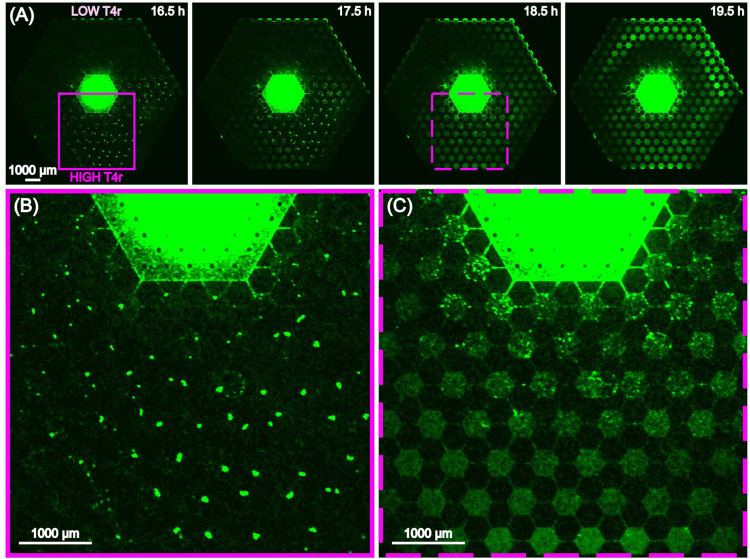
(A) Expansion of resistant *E. coli* in a gradient of T4r phage from 16.5 hours after inoculation to 19.5 hours. (B) Expanded view at 16.5 hours of the region at high T4r concentration where the bacteria transiently form small clusters. (C) Rapid dissolution of the bacterial aggregates and movement to nanoslits.

Inoculation of the resistant bacteria into the same complex ecology (with T4r gradient) that resulted in the evolution of the resistant strain shows a more nuanced picture of the changed growth properties of the mutants than the 96 wellplate experiments of Fig. 4. Fig. 6 and supplementary movie 2 shows the rather complex response of the mutant strain in a T4r gradient and a set of connected habitats. At the low T4r titer side as expected we see movement to the nanoslits and growth, Fig. 6A. At the intermediate and high titer concentrations, we see a transient aggregation of the bacteria into small clusters within each microhabitat for a period of several hours, followed by the dissolution of the clusters and uniform growth even at high titers of T4r (Fig. 6B, C). The chemotactic waves - that are typical for *E. coli* - were also present under such circumstances.

## 4. Mutations rates and evolution concepts

The ultimate observance of phage T4r resistant bacteria has three possibilities: (1) What are the odds that the initial inoculate of bacteria in the test tube had preexisting T4r resistant bacteria?; (2) What are the odds that the test tube from which we drew *N*_*o*_ bacteria spontaneously evolved resistant bacteria in the absence of T4r and we inoculated resistant bacteria into the device?; (3) What are the odds that during the time that the bacteria grew in our device that a mutation occurred that gave rise to resistance due to contact with phage? These three questions get to the heart of the conflict between viewing all resistance as being spontaneously derived without exposure (“Darwinian”) or viewing resistance emergence as a sum of Darwinian spontaneous evolution and stress-induced mutations (“Lamarckian”).

Holmes et al. [6] have carried out an extensive analysis of the original Delbrück-Luria experiments and have included both spontaneous (“Darwinian”, Θ_*D*_) and in stress response (“Lamarckian”, Θ_*L*_) mutation rates in what they called a Composite model which included both mutation mechanisms. Based on their calculations the pure Lamarckian model is inconsistent with the experimental data (as expected), but the Composite model (containing both mechanisms) and the pure Darwinian model fit equally well. As Holmes et al. point out, the dynamics of such an evolving and reproducing system is quite complex, there exist no analytical solutions. In the followings we give an estimation of mutation rates in our own experimental setup.

We can make some simple assumptions to guess how many pre-existing mutants were in our original inoculation. In a “worst case” scenario, assume that a single but specific basepair mutation suffices to give phage resistance (but see below, the number is larger). If we start with *N*_*o*_ bacteria and end with *N* bacteria, the total number of bacteria that ever lived is 2*N* − *N*_*o*_. In the absence of stress (no phage contact) the spontaneous mutation rate is Θ_*D*_ ~ 10^−9^ basepairs/generation [35]. This means that for a single resistant mutation (as opposed to any mutation), in the first generation Θ_*D*_*N*_*o*_ are resistant, and *N*_*o*_(2 − Θ_*D*_) are sensitive. After a total of *G* generations we will have [*N*_*o*_(2 − Θ_*D*_)]2^*G*−1^ sensitive mutants, and (Θ_*D*_*No*)2^*G*−1^ resistant ones. It is possible to then continue down this line from generation to generation, in each generation of *N*_*o*_2^*g*−1^ total bacteria we again get *θ*_*D*_*N*_*o*_2^*g*‒1^ resistant mutants, assuming that the pool of sensitive bacteria has not changed greatly due to Θ_*D*_ << 1. Under the assumption of a relatively unchanged pool for mutants from generation to generation, we sum to get the total number of resistant mutants *N*_*r*_ starting with *N*_*o*_ sensitive mutants in G generations:

(1) $$N_{r}\sim G\Theta_{D}N_{o}2^{G-1}$$

This yields for the fraction ℱ of bacteria that have resistance due to purely spontaneous mutations Θ_*D*_ after G generations:

(2) $$ℱ\sim \frac{G\Theta_{D}N_{o}2^{G-1}}{N_{o}2^{G-1}}G\Theta_{D}$$

Thus, only *G*Θ_*D*_*N*_*o*_ bacteria would have been expected to be already resistant due to spontaneous (Darwinian) mutations. In our case *G* ~ 24 for the initial expansion, the number of expected already resistant bacteria in the initial inoculation of *N*_*o*_ ~ 10^4^ bacteria is vanishingly small, while we saw resistance emerge with prolonged phage exposure (unlike Delbrück Luria experimental protocol) in each experiment.

We can attempt a very rough estimate of the stress-induced (Lamarckian) mutation rate Θ_*L*_ in a similar way, again in the assumption that only 1 mutation can give rise to resistance (which is not the case, as gathered from our sequencing results). In the chip we incubated against T4r phage for approximately 15 hrs before the growth of an insensitive population was observed, or roughly 30 generations, starting with *No* ~ 10^4^. Assuming we see at least 1 resistant mutant in 30 generations, this yields the mutation rate Θ_*L*_:

(3) $$\Theta_{L}\sim \frac{1}{30\times 10^{4}}\sim 10^{-5}$$

This is a vastly higher rate than the Darwinian rate Θ_*D*_. However, it is not unprecedented given the role the SOS response and presumable hypoxic conditions in biofilms that we observe can provide in accelerating mutation frequencies [56].

Our experiments ran for 3 days before bacteria were collected from the device and further analyzed. Therefore, it is also worth mentioning the stress-induced mutation rate calculated in the same way for 72 hours (roughly 140 generations). That is ~ 10^−6^, which is still order of magnitudes higher than the spontaneous Θ_*D*_ mutation rate.

## 5. Discussion

Bacteria and phages coexist in nature performing a continuous co-evolutionary co-existence. As part of this evolutionary co-existence, bacteria have evolved different mechanisms to survive phage infections and the phage have evolved mechanisms not to destroy their hosts, whom they need.

The natural environment of the perhaps somewhat onesided but still symbiotic relation between phage and bacteria [57] is not the well-stirred chemostat of the microbiology laboratory [58]. Spatial and temporal inhomogeneities in the distribution of stress factors (selection pressure) have a profound impact on evolutionary processes, changing population numbers and the invasion of resistant strains into sensitive ones. Understanding how the environment and ecology can change both mutation rates and selection dynamics is important to properly describe evolutionary processes [59].

Typically bacterial strategies for survival in complex environments involve both phenotypic and genetic adaptations. In terms of genetic adaptation, ordinarily *E. coli* have a remarkably faithful DNA replication system with an error rate on the order of 10^−9^/bp/generation [60] in the absence of stress. In our previous antibiotic evolution experiments we triggered the highly error-prone DNA replication response of the SOS system in *E. coli* [61], driven by the appearance of singlestranded DNA due to gyrase A blockage by ciprofloxacin, to form the driver of rapid evolution. The formation of filamentous bacteria [62] was an indication that this heightened evolution acceleration is occurring [63].

For phage T4r there is no corresponding SOS response to provide and evolution exit to stress via enhanced mutation rates. Apparently, at least for T4r, phage exposure alters different phenotypes, including in our case transient biofilm formation. Growing evidence shows that bacteriophages can modulate biofilm development [64], but these studies mostly deal with lysogenic phage and not virulent ones. The influence of virulent phages on biofilm formation is more limited and mostly relates to the study of the eradication of biofilms with high-phage titers.

The genetic response of bacteria in our device was heterogeneous and varied from run to run, although there were unifying themes. For example, observed mutations in the RcsC system are plausible since the Rcs signalling system controls the transcription of numerous genes involved in biofilms, e.g. genes that are involved in colanic acid capsule synthesis, production of cell surface-associated structures (flagella, LPS, fimbriae), biofilm formation and cell division [65]. RcsC is an important part of this system, functions as a membraneassociated protein kinase, and it is activated during growth on a solid surface [66]. In *E. coli*, Poranen et al. observed upregulation of genes necessary for capsule synthesis (RcsA) at 10 minutes post-infection [67]. Exopolysaccharide induction and colonic acid synthesis is a typical defense mechanism that protects the bacterial population but seems to be jettisoned once resistance is developed.

In our experiments, the CRISPR-Cas system of E. coli did not turn on, which is in good agreement with the literature. Efficient protection against phages by CRISPR/CAS-E has not been observed in the non-manipulated wild-type E. coli K12 strain. The Cascade (CRISPR-associated complex for antiviral defence) genes form an operon whose expression is repressed under normal laboratory growth by the transcriptional regulator H-NS (histone-like nucleoid structuring protein) [22,23]. It is not known what physiological circumstances are needed to turn it on.

There is a movement now to re-develop phage therapy [68] as an alternative to antibiotics because of the emergence of “super-bugs” with Multi-antibiotic resistance [69]. However, we would caution that because of our observed fast emergence resistance in a structured environment designed to award mutants with fitness-enhancing adaptions, successful therapeutic applications must be carefully designed to account for deep changes in evolution dynamics that can occur in complex environments [70] and the passage of time to phage exposure [71].

## 6. Conclusions

The widespread emergence of antibiotic-resistant bacteria has led to a resurgence in interest in phage therapy to control bacterial infections [72]. However, our study shows that the rapid emergence of stress-induced phage resistance can also occur, as perhaps one would expect given the long co-existence of phage with bacteria. We show that while our mutant resistant *E. coli* have expected mutations in the T4 OmpC binding site, they also have mutations in genes associated with biofilm mechanics, which based on our observations of the emergence of resistance in our microecologies is expected. Alas, the mutagenic mechanism driving this resistance acceleration in our hands remains unknown.

## Figures and Tables

**Table 1.** Summary of the relevant mutations found in resistant mutants extracted from the microfluidic device after exposure to a gradient of T4r

Sample	Gene	Description	Mutation
mutant1	*ompC*	outer membrane protein C	MC (8bp)
	*csgB*	minor curli subunit	MC (526bp)
	*csgD*	CsgBAC operon	MC (155bp)
mutant2	*csgB*	minor curli subunit	MC (482bp)
	*csgD*	CsgBAC operon	MC (155bp)
	*rfaP*	outer membrane lipopolysaccharide core	MC (218bp)
	*rcsC*	sensor histidine kinase RcsC	T →G (CTG →CGG
mutant3	*ompC*	outer membrane protein C	+T (TAT →TTT)
	*rfaP*	outer membrane lipopolysaccharide core	MC (218bp)

## Data Availability

The data generated in this study are available from the corresponding author upon reasonable request. The raw sequences are available at the European Nucleotide Archive (ENA) database under the study number PRJEB73316.
